# The probiotic and immunomodulation effects of *Limosilactobacillus reuteri* RGW1 isolated from calf feces

**DOI:** 10.3389/fcimb.2022.1086861

**Published:** 2023-01-12

**Authors:** Kailang Huang, Weibing Shi, Bin Yang, Jiakun Wang

**Affiliations:** Institute of Dairy Science, College of Animal Sciences, Zhejiang University, Hangzhou, China

**Keywords:** *Limosilactobacillus reuteri*, probiotic, genome analysis, antibacterial activity, immunomodulation

## Abstract

**Introduction:**

*Limosilactobacillus reuteri* is a gut symbiont with multiple remarkable beneficial effects on host health, and members of *L. reuteri* are valuable probiotic agents. However, *L. reuteri* showed obvious host specificity.

**Methods:**

In our study, a novel *L. reuteri* RGW1 was isolated from feces of healthy calves, and its potential as a probiotic candidate were assessed, by combining *in vitro*, *in vivo* experiments and genomic analysis.

**Results and discussion:**

RGW1 was sensitive to all the antibiotics tested, and it did not contain any virulence factor-coding genes. This isolate showed good tolerance to acid (pH 3.0), 0.3% bile salt, and simulated gastric fluid. Moreover, this isolate showed a high hydrophobicity index (73.7 ± 4.6%) and was able to adhere to Caco-2 cells, and antagonize *Escherichia coli* F5. Treatment of LPS-induced mice with RGW1 elevated TGF-β and IL-10 levels, while RGW1 cell-free supernatant (RCS) decreased TNF-α levels in the sera. Both RGW1 and RCS increased the villus height and villus height/crypt depth ratio of colon. Genomic analysis revealed the mechanism of the probiotic properties described above, and identified the capacity of RGW1 to biosynthesize L-lysine, folate, cobalamin and reuterin *de novo*. Our study demonstrated the novel bovine origin *L. reuteri* RGW1 had multiple probiotic characteristics and immunomodulation effects, and provided a deeper understanding of the relationship between these probiotic properties and genetic features.

## 1 Introduction

Probiotics are defined by the FAO and WHO as “live microorganisms that, when administered in adequate amounts, confer a health benefit on the host” (Hill et al., 2014). Various studies have demonstrated the health-promoting effects of probiotics with mechanisms including anti-pathogen activity and immunomodulation. To achieve these effects, probiotics should exhibit characteristics such as acid, bile and digestive enzyme tolerance, which allows them pass through the stressful environment of the host’s upper gastrointestinal tract, survive in the gut, and adhere to the intestinal wall for immunomodulation. In addition, probiotics must be safe for humans or animals, with the most important concerns being their virulence potential and the possibility to act as a reservoir for mobile antimicrobial resistance genes. Most importantly, probiotics should provide one or more benefits to host health, such as the prevention of enteric infections, diarrhea or inflammatory bowel disease.

*Limosilactobacillus reuteri* is a well-known probiotic species that naturally colonizes the gastrointestinal tract of a wide range of vertebrates. This species displays multiple beneficial effects on host health, including anti-pathogenic capacity, the production of vitamins and amino acids, modulation of host immune responses and promotion of gut mucosal integrity. However, the effects of probiotics on host health are species-, dose- and disease-specific (Aureli et al., 2011). Furthermore, *L. reuteri* has developed fundamentally different ecological strategies for different hosts and exhibits obvious host specificity (Walter, 2008; Frese et al., 2011; Duar et al., 2017). Therefore, isolating and characterizing more novel *L. reuteri* strains especially from relatively unexplored ecological niches, still makes sense to meet the increasing desire to promote human or animal health naturally.

In addition to meeting the prerequisites for being considered as a potential probiotic (described above), additional probiotic properties have been described by Mu et al. (2018), for example, vitamin B9, B12 and exopolysaccharide biosynthesis. Whole genome sequence analysis helps to efficiently identify these potential properties and enhance understanding of the relationships between genotypic and phenotypic profiles. Since previous research have reported the genomes of *L. reuteri* isolates originated from humans, rodents and poultry (Oh et al., 2010; Frese et al., 2011; Duar et al., 2017), there has been a relative lack of research into bovine *L. reuteri* strains and their genome. In this study, a new *L. reuteri* strain originated from bovine, named *L. reuteri* RGW1, was isolated and identified, and its potential probiotic traits were characterized. Additionally, the draft genome sequence of this strain was present, and probiotic-associated genes were identified.

## 2 Materials and methods

### 2.1 Isolation and culture of LAB strains

Fresh fece samples were collected from three one-month-old healthy calves immediately after morning feeding (Zhejiang Yijing Ecological Animal Husbandry Co., Ltd., Shaoxin, China). The feces were fully homogenized, and cultured using anaerobic Man, Rogosa and Sharpe (MRS) broth for 24 h. Then the culture broth was serially diluted 10-fold using sterile anaerobic PBS, spread on MRS agar and incubated at 37 °C anaerobically. After incubation for 48 h, single white colonies were selected and purified by restreaking on MRS agar plates. Their taxonomic status was determined using the 16S rRNA gene sequence with the primers 27F (5′-AGAGTTTGATCCTGGCTCAG-3′) and 1492R (5′-TACGGCTACCTTGTTACGACTT-3′), and compared to those in the National Center Biotechnology Information (NCBI) database using the nucleotide BLAST tool. The phylogenetic tree was constructed using MEGA-X software by the neighbor-joining method, and visualized using iTOL (Letunic and Bork, 2021). The *L. reuteri* isolate was Gram-stained, and its bacterial morphology was observed by scanning electron microscopy (Hitachi SU-8010, Japan). The isolate was preserved at -80 °C within 20% sterile glycerol dilution for further use.

### 2.2 *In vitro* studies on probiotic characteristics

#### 2.2.1 Tolerance to acid, bile salt and simulated gastric fluid

The growth kinetics of *L. reuteri* RGW1 was determined by measuring the optical density every hour at 600 nm. Acid or bile salt survivability was determined in MRS broth. Briefly, *L. reuteri* RGW1 was cultured overnight in anaerobic MRS broth at 37 °C, then the bacteria was incubated in MRS broth (2×10^6^ CFU/mL) at different pH values (7.0, 5.0, 4.0 and 3.0) or with different concentrations (0%, 0.1%, 0.3% and 0.5%) of bile salt at 37°C for 3 h, respectively. Tolerance to simulated gastric fluid of *L. reuteri* RGW1 were examined following the method described by Zárate et al. (2000) with minimal modification. Briefly, overnight-cultured RGW1 cells were incubated in the simulated gastric fluid (2×10^6^ CFU/mL, 3 g/L Pepsin, pH 2.5) at 37 °C for 6 h. The viability was enumerated by colony counts (CFU/mL) on MRS agar plate. Survivability (%) was calculated by the following formula: (final viability/initial viability) × 100. The assay was carried out in three parallel experiments.

#### 2.2.2 Hydrophobicity

Hydrophobicity was determined following the procedure of Nagpal et al. (2010) with minor modification. Briefly, overnight-cultured bacterial cell suspension was mixed with n-hexadecane and vortexed for 2 min. The absorbance of aqueous phase at 600 nm was measured as A0, and the mixture was placed at 37 °C for 1 h. Then the absorbance of aqueous phase at 600 nm was measured as A1. The hydrophobicity (%) was calculated with the following formula: (1-A1/A0) × 100.

#### 2.2.3 Adhesion to Caco-2 cells

Human colorectal adenocarcinoma cell line Caco-2 cells was used to examine the cellular adhesion of *L. reuteri* RGW1 as described previously (Jacobsen et al., 1999). Briefly, Caco-2 cells were seeded in 12-well plates to grow to approximately 80% confluency. A 100 µL of suspension of RGW1 at concentration of 10^8^ CFU/mL was added to each cell well, and then incubated at 37 °C in a 5% CO_2_ atmosphere. After 2 h incubation, the medium was removed, and the bacterial cells were washed five times with sterile PBS to remove the nonadherent bacteria. Then the cell monolayers were fixed in a 65 °C incubator, stained with safranin O solution and observed under a microscope.

#### 2.2.4 Antibiotic susceptibility

Antibiotic susceptibility was determined by the disc diffusion method following the procedure of Charteris et al. (1998) with minimal modification. Two hundred microliters of RGW1 cells at a concentration of 10^8^ CFU/mL were coated on MRS agar plates. After the surface was completely dried, the antibiotic discs with ampicillin (10 μg), amoxicillin (30 μg), chloramphenicol (30 μg), cefazolin (30 μg), erythromycin (15 μg), tetracycline (30 μg), or penicillin G (10 μg), respectively were placed on the center of the plate to dispense antibiotic. Then the plates were incubated anaerobically at 37 °C for 24 h. The diameter of inhibition zone was measured, and the results were expressed in terms of resistance, moderate susceptibility, or susceptibility, according to the interpretative standards detailed in **Supplementary Table S1**.

#### 2.2.5 Virulence genetic amplification

As reported by Casarotti et al. (2017) and Nagpal et al. (2018), virulence genes including the adherent virulence factors *esp* (*Enterococcal* surface protein), *efaAfs* (cell wall adhesins), *asa* (aggregation substance), and *ace* (adhesion of collagen), the secretory virulence factors *gelE* (gelatinase), *cylA* (protein for activation of cytolysin), *hyl* (hyaluronidase) and the biogenic amine production factors *hdc* (histidine decarboxylase), *tdc* (tyrosine decarboxylase) and *odc* (ornithine decarboxylase), were detected using PCR. The amplified products were resolved by electrophoresis on a 1% agarose gel. Detailed primer information for these virulence genes is shown in **Supplementary Table S2**.

#### 2.2.6 Antibacterial activity

The antibacterial activity was determined by the well agar diffusion method and coculture experiment. *Escherichia coli* F5 (BNCC-125787, also known as K99) was purchased from BeNa Culture Collection (BNCC), and cultured and enriched using Luria-Bertani (LB) broth. For well agar diffusion, briefly, overlaid a petri dish with soft LB agar containing 10^8^ CFU/mL of *E. coli* F5. After solidification, the wells were punched with Oxford cups and seeded with 150 µL of RGW1 cells (10^8^ CFU/mL). After incubation at 37 °C anaerobically for 16 h, the diameter of inhibition zones was measured. To identify the effective antibacterial compounds, the bacteria-free supernatant of RGW1 (10^8^ CFU/mL) was pretreated by pH adjustment, proteinase K or heating, respectively. Briefly, for pH adjustment, the pH value of supernatant was adjusted to 7.0 using a 1.0 mol/L NaOH solution, then the supernatant was filtered with 0.22 μm filter membrane. For heating pretreatment, the supernatant was heated in a 70 °C water bath for 1 h. When the supernatant was cooled to room temperature, the supernatant was filtered with 0.22 μm filter membrane. For proteinase K pretreatment, the supernatant were treated with proteinase K (0.1mg/mL) in a 37 °C water bath for 30 min. Then the treated supernatant was used to determined antibacterial activity using well agar diffusion method described above. As for the coculture experiment, RGW1 and *E. coli* F5 were cocultured in a mixture of 5 mL MRS broth and 5 mL LB broth with the initial concentration RGW1 and *E. coli* F5 cells set at 10^8^ and 10^7^ CFU/mL, respectively. RGW1 monocultured in 10 mL MRS broth at 10^8^ CFU/mL and *E. coli* F5 monocultured in 10 mL LB broth at 10^7^ CFU/mL were used as controls. All cultures were incubated at 37 °C with shaking at 180 rpm. The viable counts (CFU/mL) of RGW1 and *E. coli* F5 were enumerated using MRS agar or LB agar at 0, 8, 16, and 24 h of incubation, respectively.

### 2.3 Oral administration of LAB and sample collection in mouse study

#### 2.3.1 Animals and housing

All procedures were approved by the Animal Care and Use Committee of Zhejiang Provincial Hospital of Chinese Medicine (Hangzhou, China, 60136-38385) and were in accordance with the hospital guidelines for animal research. In the current study, a total of 27 male C57BL/6J mice (5 weeks old, 18.7 ± 0.7 g initial body weight) were purchased from Shanghai SLAC Laboratory Animal Company (Shanghai, China). After three days of adaption, the mice were randomly assigned to three groups, each group contains three cages of three mice in each (n = 9). Following the dose of administration of *L. reuteri* described in Wu et al. (2020), the three groups of mice were orally administrated (1) 200 µL/d of MRS medium for seven days (control); (2) 200 µL/d of RGW1 (10^8^ CFU) for seven days (RGW1); or (3) 200 µL/d of RGW1 cell-free supernatant for seven days (RCS), respectively. The body weight (BW) and intake of all mice were recorded daily at 9 am before oral administration. Each mouse was challenged with lipopolysaccharide (LPS, 10 mg/kg BW) on day eight. On day nine, the mice were anesthetized and sacrificed by cervical dislocation after collecting blood by eyeball enucleation, and then the intestinal tissues were collected.

#### 2.3.2 Biochemical assays of serum

The concentrations of TNF-α, TGF-β and IL-10 in the serum were determined using ELISA kits (Jianglai Biotechnology Institution, Shanghai, China).

#### 2.3.3 Hematoxylin and eosin staining of the gut

Hematoxylin and eosin (H&E) staining of intestinal tissues was performed as previously described (Zhong et al., 2022). In brief, the cecum and colon samples were fixed in 4% paraformaldehyde, waxed, and cut into 5-μm thick sections before being stained. After deparaffinization, the sections were soaked with alcohols followed by staining with H&E. The villus height and crypt length were measured using Image-Pro Software (Media Cybernetics, Inc., USA) based on photomicrographs obtained by microscopy.

### 2.4 Complete genome sequencing, assembly and annotation

RGW1 genomic DNA was extracted with the cetyltrimethyl ammonium bromide (CTAB) method (Gagen et al., 2010). The whole genome was sequenced using an Illumina NovaSeq PE150 (Beijing Novogene Bioinformatics Technology Co., Ltd). Illumina PCR adapter reads and low-quality reads (< 40 bp, N > 10 bp) from the paired-end reads were filtered in quality control step using readfq (version 10). All good quality (Q30 = 92.1%, Q20 = 97.26%) paired-end reads were assembled into a number of scaffolds using SOAP denovo (Li et al., 2008), SPAdes (Bankevich et al., 2012) and ABySS (Simpson et al., 2009). Genome component prediction was achieved using the GeneMarkS program (Besemer et al., 2001), and tRNA and rRNA were identified using tRNAscan-SE (Lowe and Eddy, 1997) and rRNAmmer (Lagesen et al., 2007), respectively. The gene functions were predicted using various databases, including NR (Non-Redundant Protein Database, version 201705), GO (Gene Ontology, version 201705), KEGG (Kyoto Encyclopedia of Genes and Genomes, version 201807), COG (Clusters of Orthologous Groups, version 20151214), TCDB (Transporter Classification Database, version 201807), and Swiss-Prot (version 201807). Carbohydrate-active enzymes were predicted by the Carbohydrate-Active EnZymes Database (version 201604). Pathogenicity and drug resistance analyses were performed using PHI (Pathogen Host Interactions, version 20180515) and VFDB (Virulence Factors of Pathogenic Bacteria, version 20180726).

### 2.5 Statistical analysis

All data are displayed as the mean ± standard error. Student’s *t* test was applied to compare two groups, and one-way ANOVA was applied to compare more than two groups. Statistical significance was declared at *P* < 0.05 unless otherwise specified. All analyses were performed using the GraphPad Prism software 9.0.0 (San Diego, CA, USA).

## 3 Results

### 3.1 Bacterial isolate and its basic identification

**Figure 1:**
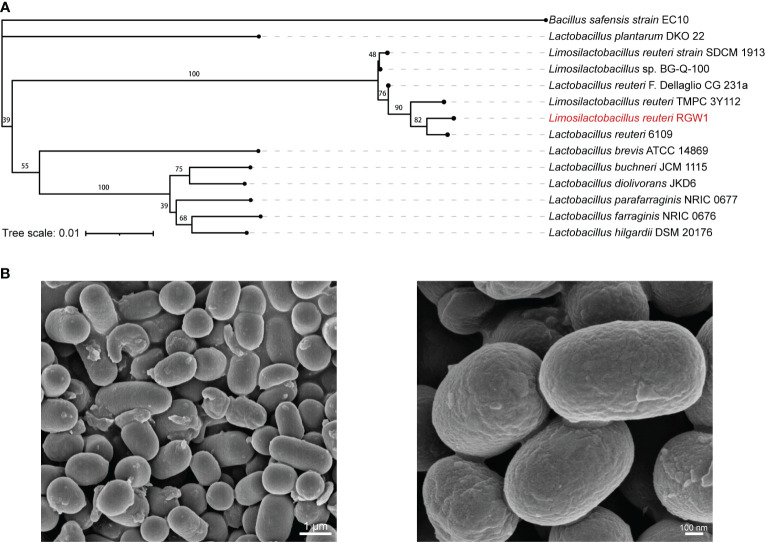
Preparation and characterization of *L. reuteri* RGW1. **(A)** Phylogenetic tree of *L. reuteri* RGW1 (in red color) based on 16S rRNA sequence. The 12 most homologous sequences in the GenBank database were selected for the construction of a phylogenetic tree, *Bacillus safensis* strain EC10 was selected as outlier bacteria. **(B)** Representative image of *L. reuteri* RGW1 using scanning electron microscope.

Potential LAB strains were isolated from healthy calf feces, and the 16S rRNA sequence-based phylogenetic tree revealed that the isolate in our study belonged to a subclade of *L. reuteri*, with more than 99% similarity to other strains within the genus (**Figure 1A**), thus, the isolate was named *L. reuteri* RGW1. The strain was identified as gram-positive and produced small white colonies on MRS agar. Scanning electron microscopy images revealed that the strain was rod-shaped (**Figure 1B**).

### 3.2 *In vitro* probiotics characteristic of RGW1

#### 3.2.1 Safety of RGW1

**Figure 2:**
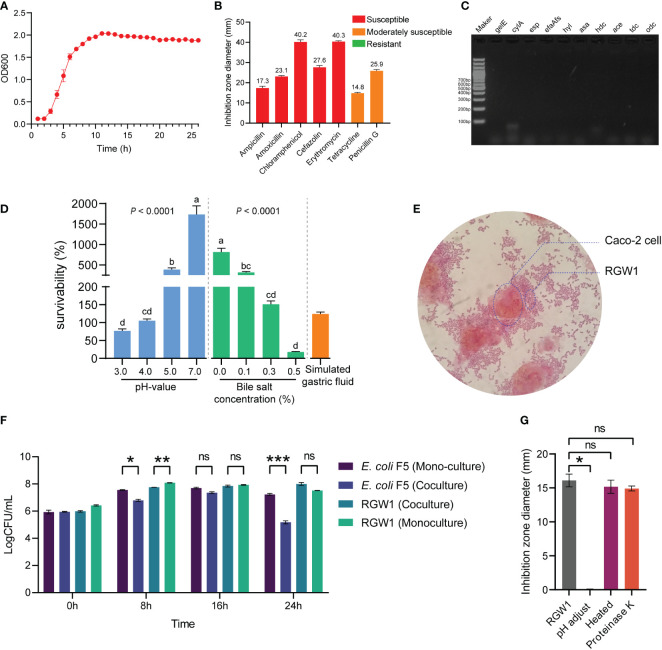
*In vitro* probiotic characteristics of *L. reuteri* RGW1. **(A)** The growth kinetics of *L. reuteri* RGW1. **(B)** Antibiotic susceptibility profile of *L. reuteri* RGW1 is indicated by the diameters of inhibition zone, the “S” represents sensitivity. **(C)** Agarose gel electrophoresis results of virulence gene PCR products. **(D)** Survivability of *L. reuteri* RGW1 at different pH values (blue), bile salt concentrations (green), and in simulated gastric fluid (orange), a-d represent different superscripted letters (P < 0.05). **(E)** Representative image of the coculture of *L. reuteri* RGW1 with Caco-2 cells stained with safranin O solution. **(F)** Monoculture or coculture of *L. reuteri* RGW1 with *E. coli* F5. Each bacterial concentration was counted at 0, 8, 16, 24 h of culture, respectively. **(G)** The anti *E. coli* F5 activity of *L. reuteri* RGW1 indicated by the diameters of inhibition zone, after different pretreatments. Values are expressed as means ± SEM. *P < 0.05; **P < 0.01; ***P < 0.001; ns, not significant.

The growth kinetics of RGW1 displayed the typical three phases, with lag, log, and stationary phages occurring from 0 to 2 h, 2 to 8 h, and after 8 h, respectively (**Figure 2A**). Antibiotic susceptibility tests showed that RGW1 was sensitive to ampicillin, amoxicillin, chloramphenicol, cefazolin, and erythromycin, and moderately sensitive to tetracycline and penicillin G (**Figure 2B**). Virulence genetic analysis showed that RGW1 does not contain virulence factors-coding genes, including *gelE*, *cylA*, *esp*, *efaAfs*, *hyl*, *asa*, *hdc*, *ace*, *tdc*, and *odc* (**Figure 2C**).

#### 3.2.2 Tolerance of RGW1 to acid, bile salt and simulated gastric fluid

RGW1 showed a survivability as high as 76.9 ± 5.4% at pH 3.0 for 3 h (**Figure 2D**). The strain showed good tolerance to 0.3% of bile salt (survivability as high as 151.1 ± 9.5%) after 3 h of exposure (**Figure 2D**). RGW1 had a high survivability of 124.1 ± 5.7% in simulated gastric fluid (**Figure 2D**). Accordingly, we conclude that RGW1 has the ability to survive in the host gastrointestinal tract.

#### 3.2.3 Potential probiotic properties

##### 3.2.3.1 Hydrophobicity and adhesion to Caco-2 cells

RGW1 showed a hydrophobicity index as high as 73.7 ± 4.6%, which significantly higher than *L. paralimentarius* DSM 13238 (*P* < 0.001, **Supplementary Figure 1A**). RGW1 was able to adhere to the cell surface of Caco-2 cells after 2 h coculture (**Figure 2E**).

##### 3.2.3.2 Antagonize E. coli F5 capacity

RGW1 showed antibacterial activity (inhibition zone was 19.2 ± 0.3 mm) against *E. coli* F5. The antibacterial activities were not significantly different between RGW1 and *L. paralimentarius* DSM 13238, as well as the antibacterial activities of both RGW1 and *L. paralimentarius* DSM 13238 were not affected by preheating, but was eliminated by pH adjustment (**Figure 2F**, **Supplementary Figure 1B-D**). However, the antibacterial activity of *L. paralimentarius* DSM 13238 was significantly downregulated (*P* < 0.05), while of RGW1 was not affected by proteinase K pretreatment (**Supplementary Figure 1B-D**). With the coculture of RGW1 (10^8^ CFU/mL) and *E. coli* F5 (10^7^ CFU/mL), the growth of both *E. coli* F5 and RGW1 was inhibited at 8 h, this inhibition was maintained in *E. coli* F5 but not in RGW1 after 24 h of coculture (**Figure 2G**).

### 3.3 Immunomodulatory effect of RGW1 in inflammatory mice challenged by LPS

**Figure 3:**
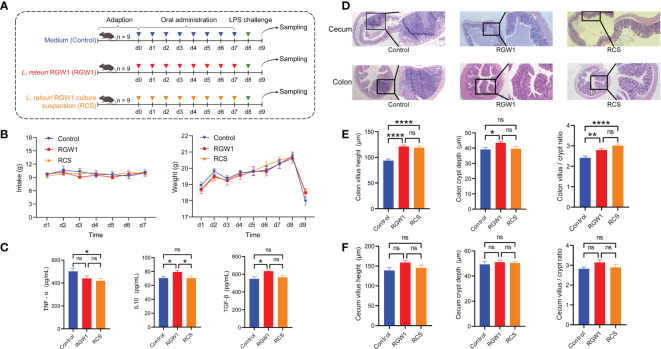
Immunomodulatory effects of *L. reuteri* RGW1 on inflammation in mice challenged with LPS. **(A)** Experimental schedule for LPS challenge and administration of *L. reuteri* RGW1 and RCS. LPS was intraperitoneally injected on day 8, *L. reuteri* RGW1 or RCS was orally administrated 7 times. **(B)** The intake and weight of mice. **(C)** The levels of serum IFN-α, TGF-β and IL-10. **(D)** Representative images of the colon stained with H&E. **(E, F)** Intestinal morphology analysis based on measurements of the villus height, crypt depth, and the ratio of villus height to crypt depth (VH/CD) in colon or cecum tissues. Values are expressed as means ± SEM. *P < 0.05; **P < 0.01; ****P < 0.0001; ns, not significant.

Mice were given RGW1 or its cell-free supernatant (RCS group) before undergoing LPS induced inflammation (**Figure 3A**). The intake and weight were not significantly different from day 1 to 7 among control, RGW1 and RCS groups, but the mouse weights significantly decreased one day after LPS challenge (**Figure 3B**). We measured the cytokines in the serum (**Figure 3C**) and found that administration of RCS significantly lowered the level of the pro-inflammatory cytokine TNF-α (*P* < 0.05), whereas administration of RGW1 significantly elevated the levels of the anti-inflammatory cytokines TGF-β (*P* < 0.05) and IL-10 (*P* < 0.05). According to the histological analysis of colon tissues (**Figure 3D**), administration of RGW1 and RCS reduced the occurrence of intestinal injury, such as a thinner mucosa, deformed crypts and swelled and shed villi. Additionally, administration of RGW1 significantly increased the villus height, crypt depth, and the villus height/crypt depth ratio of colon, while administration of RCS significantly increased the villus height and the villus height/crypt depth ratio of colon (**Figure 3E**). Both administration of RGW1 and RCS did not change the villus height, crypt depth and the villus height/crypt depth ratio of cecum **Figure 3F**).

### 3.4 Genomic features and putative probiotic properties of RGW1

#### 3.4.1 Genomic features and functional class of RGW1

**Figure 4:**
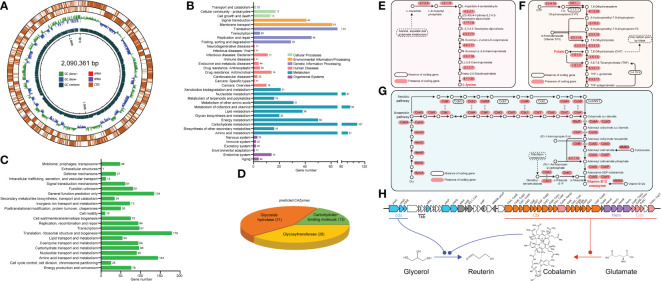
General genome features of *L. reuteri* RGW1. **(A)** Circular draft genome maps of the *L. reuteri* RGW1 chromosome. From outside to the inside: forward CDSs, reverse CDSs, GC skew, GC content. **(B)** Histogram representing the number of genes included in each KEGG pathway. **(C)** COG classes of predicted protein sequences of *L. reuteri* RGW1. **(D)** Predicted CAZymes of *L. reuteri* RGW1. **(E)** L-lysine biosynthesis pathway. **(F)** Folate biosynthesis pathway. **(G)** Cobalamin biosynthesis pathway, the sequence homology and functional equivalency between aerobic and anaerobic pathway enzymes are indicated by gray arrows. **(E-G)** were adapted from KEGG pathway. **(H)** *pdu-cbi-hem-cob* gene cluster and the proposed reuterin biosynthesis pathway in *L. reuteri* RGW1.

This draft genome of RGW1 is 2,090,361 bp in size with 39.43% GC content, and the numbers of predicted coding gene, tRNA, and 5S, 16S and 23S rRNA are 2098, 64, and 5, 1, 1, respectively (**Figure 4A**). Genome sequencing did not identify any plasmid sequences. Annotation using the NR database found that *L. reuteri* accounted for 94.9% of all organisms that showed sequence homology with the RGW1 strain. Annotation using KEGG database identified a total of 1970 genes in the genome of RGW1, most of which involved in membrane transport, translation, nucleotide, cofactors and vitamins, carbohydrates, and amino acid metabolism (**Figure 4B**). Annotation of the genome of RGW1 using COG database predicted 1417 genes, which were assigned to 22 categories of orthologous groups (COG) classes, the “translation, ribosomal structure and biogenesis” group had the highest number of genes, followed by “amino acid transport and metabolism”, “general function prediction only”, and “carbohydrate transport and metabolism” (**Figure 4C**). A total of 69 predicted carbohydrate-active enzymes (CAZymes) were identified in the RGW1 genome and divided into three subgroups, including 31 genes for glycoside hydrolases, 28 genes for glycosyltransferases, and 13 genes for carbohydrate-binding molecules (**Figure 4D**). Additionally, PHI and VFDB data annotation of the RGW1 genome did not reveal any virulence genes.

#### 3.4.2 Specific metabolism of RGW1

Genomic analysis revealed the genetic potential of RGW1 for *de novo* biosynthesis of L-lysine (**Figure 4E**), one of the essential amino acids required in the diets of animals, as well as folate (vitamin B9) (**Figure 4F**), and cobalamin (vitamin B12) (**Figure 4G**). Gene locus information is in **Supplementary Table S3-5**. The genes (Lr_GM000502 - Lr_GM000531) encoding for cobalamin biosynthesis located in *cbi-hem-cob* cluster (**Figure 4H**). Moreover, three propanediol dehydratase subunit (*pduC*, *pduD* and *pduE*) encoding genes (Lr_GM000480, Lr_GM000481, Lr_GM000482) were identified (**Figure 4H**), which have been reported to be the glycerol utilization gene candidates in the *pdu* operon and are involved in reuterin (a mixture of different forms of 3-hydroxypropionaldehyde, 3-HPA) biosynthesis, when cobalamin is present as a cofactor. Moreover, the *pdu* operon was located adjacent to the *cbi-hem-cob* gene cluster (**Figure 4H**).

#### 3.4.3 Putative probiotic feature

The coding genes involved in acid and bile tolerance, adherence, antimicrobial activity, and intrinsic defense were identified (**Table 1**) by combining the functional predictions using distinct databases as described in methods. In the genome of RGW1, amino acid/H^+^ (Lr_GM000677, Lr_GM002032), tripetide/H^+^ (Lr_GM000977), gluconate/H^+^ (Lr_GM000206, Lr_GM000385), fucose/H^+^ (Lr_GM001341), and sodium-glutamate/H^+^ (Lr_GM000634) symporters that cotransport proton and other compounds across the cellular membrane were identified. The genes coding for the Na^+^/H^+^ antiporter (Lr_GM000006, Lr_GM000042, Lr_GM001658, Lr_GM001822, Lr_GM001939) and CPA2 family monovalent cation/H^+^ antiporter (Lr_GM000790), which exchange sodium ions and protons across the membrane were also documented. F1/F0 ATP synthase is essential to maintain intracellular pH homeostasis, and the genes coding for F1/F0 ATP synthase subunits (Lr_GM001315, Lr_GM001316, Lr_GM001317, Lr_GM001318, Lr_GM001319, Lr_GM001320, Lr_GM001321, Lr_GM001322) were identified in the genome of RGW1. RGW1 harbored the genes that encode MFS transporter (Lr_GM000643, Lr_GM000660, Lr_GM000723, Lr_GM000804, Lr_GM000837, Lr_GM001128, Lr_GM001144, Lr_GM001447, Lr_GM001948, Lr_GM001976, Lr_GM001977) and choloylglycine hydrolase (Lr_GM001633), which are linked to bile tolerance. RGW1 also carries the genes encoding NADH peroxidase (Lr_GM001770, Lr_GM001291), thioredoxin (Lr_GM000347, Lr_GM001596), and thioredoxin reductase (Lr_GM000748, Lr_GM000787, Lr_GM000903, Lr_GM001006), which are associated with antioxidant capacity. RGW1 also carries various heat stress protein-coding genes, including heat shock protein Hsp33 (Lr_GM002012), heat shock protein Hsp60 (Lr_GM001766, Lr_GM001767), molecular chaperone DnaJ (Lr_GM000594), molecular chaperone DnaK (Lr_GM000595), and nucleotide exchange factor GrpE (Lr_GM000596).

**Table 1:** List of putative probiotic genes of *L. reuteri* RGW1.

Putative function	Protein class	Gene ID
Acid tolerance	Amino acid/H+ symportor	Lr_GM000677, Lr_GM002032
	Tripetide/H+ symportor	Lr_GM000977
	Gluconate/H+ symportor	Lr_GM000206, Lr_GM000385
	Fucose/H+ symportor	Lr_GM001341
	Sodium-glutamate/H+ symportor	Lr_GM000634
	Atiporter	Lr_GM000006, Lr_GM000042, Lr_GM001658, Lr_GM001822, Lr_GM001939
	F1/F0 ATP synthase	Lr_GM001315, Lr_GM001316, Lr_GM001317, Lr_GM001318, Lr_GM001319, Lr_GM001320, Lr_GM001321, Lr_GM001322
Bile tolerance	MFS transporter	Lr_GM000643, Lr_GM000660, Lr_GM000723, Lr_GM000804, Lr_GM000837, Lr_GM001128, Lr_GM001144, Lr_GM001447, Lr_GM001948, Lr_GM001976, Lr_GM001977
	Choloylglycine hydrolase	Lr_GM001633
Antioxidant	NADH peroxidase	Lr_GM001770, Lr_GM001291
	Thioredoxin	Lr_GM000347, Lr_GM001596
	Thioredoxin reductase	Lr_GM000748, Lr_GM000787, Lr_GM000903, Lr_GM001006
Heat stress protein	Heat shock protein Hsp33	Lr_GM002012
	Heat shock protein Hsp60	Lr_GM001766, Lr_GM001767
	Molecular chaperone DnaJ	Lr_GM000594
	Molecular chaperone DnaK	Lr_GM000595
	Nucleotide exchange factor GrpE	Lr_GM000596
Antimicrobial	Reuterin	See **Figure 4G–H**

#### 3.4.4 Molecular determinants of host-bacteria interactions

A total of 19 surface protein-coding genes were identified from the genome of RGW1 (**Table 2**). RGW1 contains the genes encoding cell surface proteins involved in adhesion, including CBS domain-containing protein (Lr_GM001233), elongation factors (Lr_GM000593, Lr_GM000622, Lr_GM000958, Lr_GM001688, Lr_GM001713, Lr_GM001782, Lr_GM001875, Lr_GM001965), LPxTG-motif cell wall anchor domain proteins (Lr_GM000196, Lr_GM000922, Lr_GM000933, Lr_GM001344, Lr_GM001561, Lr_GM001672, Lr_GM001699), fibronectin/fibrinogen-binding protein (Lr_GM001088), and peptidoglycan-binding proteins (Lr_GM001332, Lr_GM001975). These surface proteins are involved in colonization by binding to epithelia, epithelial cells, or mucus. RGW1 encodes genes involved in lipoteichoic acid (LTA), peptidoglycan (PG), and exopolysaccharide (EPS) biosynthesis, including LTA biosynthesis proteins (Lr_GM000913, Lr_GM001244, Lr_GM001293), LTA exportation protein (Lr_GM001113), LTA acyltransferase (Lr_GM001242), PG biosynthesis regulator (Lr_GM001437), and EPS biosynthesis proteins (Lr_GM000802, Lr_GM001532, Lr_GM001751), which can activate different pattern recognition receptors present in the epithelial cells and immune cells.

**Table 2:** Molecular determinants of host-bacteria interaction associated genes.

Putative function	Protein class	Gene ID
Surface protein-encoding genes	Enolase	Lr_GM001934
	CBS domain-containing protein	Lr_GM001233
	Elongation factor	Lr_GM000593, Lr_GM000622, Lr_GM000958, Lr_GM001688, Lr_GM001713, Lr_GM001782, Lr_GM001875, Lr_GM001965
	LPXTG-motif cell wall anchor domain protein	Lr_GM000196, Lr_GM000922, Lr_GM000933, Lr_GM001344, Lr_GM001561, Lr_GM001672, Lr_GM001699
	Fibronectin/fibrinogen-binding protein	Lr_GM001088
	Peptidoglycan-binding protein	Lr_GM001332, Lr_GM001975
Immunomodulation	Lipoteichoic acid (LTA) biosynthesis protein	Lr_GM000913, Lr_GM001244, Lr_GM001293
	Lipoteichoic acid (LTA) exportation protein	Lr_GM001113
	Lipoteichoic acid (LTA) acyltransferase	Lr_GM001242
	Peptidoglycan (PG) biosynthesis regulator	Lr_GM001437
	Exopolysaccharide (EPS) biosynthesis protein	Lr_GM000802, Lr_GM001532, Lr_GM001751

## 4 Discussion

In this study, one new *L. reteuri* strain named RGW1 was isolated from calf feces and identified based on 16S rRNA gene sequencing. Combining genomic analysis with experimental studies, we revealed a variety of probiotic effects of *L. reteuri* RGW1, including resistance to harsh environments, anti-pathogenic activity, adhesion to host cells and immunomodulation. We also identified numerous probiotic-associated genes, which could at least partially explain its probiotic properties. Our study extends the probiotic library with the bovine origins and provides genotypic clues for the probiotic characteristics of RGW1.

### 4.1 Safety assessment

Antibiotic susceptibility and virulence factors are important indicators of safety. Probiotics must not participate in undesirable antibiotic resistance gene transfer cascades *in vivo*. Thus, we surveyed the susceptibility of RGW1 to seven antibiotics: ampicillin, amoxicillin, cefazolin, and penicillin G are inhibitors of cell wall synthesis, and chloramphenicol, erythromycin and tetracycline are inhibitors of protein synthesis (Charteris et al., 1998). The virulence genes tested in our study encode (1) adherent virulence factors (*esp*, *efaAfs*, *asa* and *ace*) which aid the bacterium in adhesion and evasion of the host cell; (2) secretory virulence factors (*gelE*, *cylA*, *hyl*) which help the bacterium wade through the innate and adaptive immune response mounted within the host; and (3) decarboxylase enzymes (*hdc*, *tdc* and *odc*), which act as biogenic amine production factors involved in biogenic amine production. The overproduction of biogenic amines has been proved to be toxic, causing symptoms such as diarrhea, food poisoning, vomiting, sweating or tachycardia (Özogul and Hamed, 2018; Wójcik et al., 2021). RGW1 is not resistant to any antibiotics tested in our study and contains none of the virulence genes tested, these results demonstrate that RGW1 is safe for application.

### 4.2 Tolerance

Growth at a pH of 3.0 is usually considered optimal for a successful probiotic to survive, due to the buffering impact of ingested food that raises the pH in the stomach environment to 3.0 (Liu et al., 2013). *L. reteuri* RGW1 showed a survivability of more than 100% at pH 3.0. Such property coincides with the genome of RGW1, which contains a series of acid resistance genes coding for symporters, Na/H^+^ antiporters and the F1/F0 ATP synthase subunits. The first two protein familys play important roles in transporting proton, and the latter has a synthase function in the ATP synthesis coupled with proton transport. Previous studies have demonstrated that overproduction of ATP synthase subunit beta is associated with acid resistance in *L. pentosus* (Pérez Montoro et al., 2018b). In addition to the stomach environment, it is also essential for a probiotic to display tolerance to small intestinal transit, due to the antimicrobial impact of bile salt (Charteris et al., 1998). The MFS (major facilitator superfamily) transporter is a membrane transporter superfamily that can facilitate the movement of small solutes on the cell membrane and expel intracellular antibiotics (Pao et al., 1998; Langton et al., 2005). Three MFS genes have been reported to be bile salt exposure associated genes (Ruiz et al., 2012). Choloylglycine hydrolase (bile salt hydrolase) is essential for bile acid homeostasis in the host and represents a vital contribution of the gut microbiome to host health. Bile salt hydrolase has been reported in *Lactobacillus* (Long et al., 2017). In our study, RGW1 displayed a degree of tolerance to 0.3% bile salt, and this capacity can be explained by the presence of genes coding for MFS transporters and one bile salt hydrolase in the genome of RGW1. In addition, *L. reteuri* RGW1 survived well in the simulated gastric fluid. These *in vitro* results with genomic analysis indicate the potential of *L. reteuri* RGW1 in nutritional and clinical applications.

### 4.3 Adhesion

Adhesion of probiotics to the host epithelium is thought to increase host-bacterial interactions, which are linked to both competitive exclusion of pathogens and immunomodulation mechanisms (Hymes et al., 2016; Ku et al., 2016). Previous study has revealed the correlation between hydrophobicity and adhesion of probiotic bacteria (Pan et al., 2006). In our study, the high hydrophobic index of RGW1 was consistent with its strong adhesion to Caco-2 cells. *L. reteuri* RGW1 encodes most of the known adhesion factors, including elongation factors, an LPxTG-motif protein, fibronectin/fibrinogen-binding proteins, peptidoglycan-binding proteins, and enolase. Muscariello et al. (2020) reviewed the mechanisms by which these adhesion proteins in *Lactobacillus* are involved in adhesion to the host cell, mucin, or the intestinal epithelia. One possible mechanism for adherence is through binding to the mucus layer. Elongation factors (EFs) function in “translation, ribosomal structure and biogenesis” according to the GO function annotation; however, some EF proteins have been shown to be involved in bacterial adhesion. For example, EF-Tu was demonstrated to be a mucin adhesion factor identified at the surface of some *Lactobacillus* species (Dhanani and Bagchi, 2013; Nishiyama et al., 2013; Du et al., 2022); furthermore, EF-GreA was reported to be an adhesion biomarker in *L. pentosus* using immobilized mucin model (Pérez Montoro et al., 2018a). In addition to EF-Tu and EF-GreA, other EF coding genes were identified in the RGW1 genome. LPxTG-motif proteins are a type of precursor protein that carries a conserved LPxTG-motif at the C-terminus, these proteins were reported to promote the adhesion of *L. rhamnosus* GG to the intestinal mucosa (von Ossowski et al., 2011) and the intestinal survival rate and adhesion characteristics of *L. reuteri* (Xu et al., 2022). In addition to adhering to the mucus layer, another possible adherent mechanism is binding to a variety of proteins present in the extracellular matrix (ECM) of intestinal epithelial cells, such as fibronectin, collagen, and peptidoglycan. RGW1 carries one gene coding for a fibronectin/fibrinogen-binding protein and two genes coding for peptidoglycan-binding proteins. Besides, the genome of RGW1 contains the gene coding for enolase, which was shown to mediate the adhesion of *L. casei* BL23 to fibronectin and collagen (Muñoz-Provencio et al., 2011) and *L. plantarum* to collagen (Salzillo et al., 2015). Enolase is also involved in the immunostimulation of Caco-2 cells and biofilm development (Vastano et al., 2016). The presence of these protein coding genes in the RGW1 genome helps to explain the strong adhesion of this isolate to Caco-2 cells. Because the adhesion can be host-dependent, further testing will be required to assess more RGW1 adhesion characteristics of with different host.

### 4.4 Inhibition of pathogens

The anti-pathogenic property of probiotics is an important benefit. Here, the anti-pathogenic effects of RGW1 were tested against enterotoxigenic *E. coli* F5, one of the pathogens that causes diarrhea in calves. *L. reteuri* RGW1 showed inhibitory activity against *E. coli* F5. Combining with the properties that RGW1 could adhere to Caco-2 cell, this result suggested the potential probiotic characteristics *in vivo* of enhancing colonization resistance thus preventing animal severe diarrhea caused by pathogen. Among the treatments to the RGW1 suspension that we tested, only the adjustment of pH reduced its inhibitory activity. These results indicated that the antimicrobial compounds of RGW1 are tolerant to heat therefore are not be peptides or proteins. Reuterin is a well-known antimicrobial compound, which can be produced and excreted by most *L. reuteri* strains. The synthesis of reuterin is mediated by glycerol dehydratase, which catalyzes the conversion of glycerol to 3-HPA. Previous study revealed that propanediol dehydratase coding genes (*pduC*, *pduD*, *pduE*) act as glycerol utilization candidates and encode the glycerol dehydratase activity in *L. reuteri* JCM 1112(T) (Morita et al., 2008). Both propanediol and glycerol dehydratase enzymes require cobalamin as cofactor. Cobalamin biosynthesis requires more than 30 enzymatic steps, *via* aerobic or anaerobic pathways. Herein, we found that RGW1 possesses all of the genes involved in the anaerobic pathways, while *cobG*, *cobF* and *cobNST* involved in the aerobic pathways were absent. These results indicated that RGW1 might synthesize cobalamin in an anaerobic environment rather than aerobically. The gene sets for cobalamin synthesis (*cbi*, *cob*, and *hem*) in RGW1 are located adjacent to the *pdu* operon, and the structure of this gene cluster probably reflects the requirement for glycerol dehydratase activity. Not surprisingly, the strong anti-pathogenic effects of *L. reuteri* strains were associated with the presence of the *pdu-cbi-cob-hem* gene cluster (Lee et al., 2017). Genomic analysis further supports the potential role of RGW1 reuterin in anti-microbiota. While RGW1 showed good anti *E. coli* F5 activity and the reuterin has a good antibacterial broad-spectrum. The anti-pathogen activities of RGW1 against both gram-positive and negative pathogen, such as *Salmonella*, *Staphylococcus*, *Streptococcus*, should be tested in the future, as well as the *in vivo* experiments will be conducted.

### 4.5 Immunomodulation

Feeding mice RGW1 or its cell free supernatant did not affect their weight or intake. We also investigated its immunomodulation properties using mouse module of inflammation induced by the gram-negative bacterial LPS, which is known to induce acute inflammatory responses in the host (Guijarro-Muñoz et al., 2014). Previous research reported that *L. reuteri* CRL1101 downregulated the levels of cytokines (IL-1β, IL-6 and TNF-α) in the sera of mice stimulated with LPS (Juarez et al., 2013), and *L. reuteri* ZJ617 and ZJ615 downregulated TNF-α in the sera of mice stimulated with LPS (Gao et al., 2016). Consistent with these findings, the metabolites of *L. reuteri* RGW1 exerted anti-inflammatory properties by downregulating TNF-α in the sera significantly, while live *L. reuteri* RGW1 downregulating TNF-α numerically. This phenomenon might be explained by that some compounds in the surface of live *L. reuteri* RGW1 could stimulate host, thus slightly altering the immune status. As evidenced by Spindler et al. (2022), who revealed that the gut commensals could also stimulate phylogenetically defined innate immune responses, including TNF-α expression.

Exopolysaccharide (EPS) is a self-produced extracellular component for biofilm formation (Salas-Jara et al., 2016) that has anti-inflammatory activity (Ryan et al., 2019). Lipoteichoic acid (LTA) is the major microbe-associated molecular pattern of gram-positive bacteria and the agonist of Toll-like receptors (Claes et al., 2012). LTA of *L. reuteri* was reported to alleviate inflammatory responses, as evidenced by the altered levels of the inflammatory cytokine TNF-α, IL-6, and IL-10 (Lu et al., 2022). PG are more likely to activate NOD-like receptors (Wolf and Underhill, 2018). Both Toll-like and NOD-like receptors are pattern recognition receptors that carefully regulate downstream cytokine secretion, and are therefore associated with the regulation of intestinal immune conditioning and responses that limit bacterial burden and prevent excessive inflammatory responses (Abraham et al., 2022). The genes coding for EPS, LTA, and PG biosynthesis have been identified in the genome of RGW1, suggesting that RGW1 may alleviate inflammatory responses through these molecules. Folate (vitamin B9) is essential for several metabolic preprocesses involved in the transfer and formation of one-carbon (C1) units, and C1 metabolism is critical for purine and thymidylate synthesis, methylation reactions and the metabolism of several amino acids. Furthermore, folate synthesis and metabolism by some specific *L. reuteri* strains (such as *L. reuteri* 6475) were also demonstrated to contribute to the suppression of inflammation (Thomas et al., 2016; Röth et al., 2019). In the current study, all genes for *de novo* biosynthesis of folate in the presence of vitamin B10 were identified in the genome of RGW1, as a rare exception, vitamin B10 can be synthesized by the genera *Lactococcus* and *Streptococcus* (Rossi et al., 2011). These results indicate that RGW1 has the potential to biosynthesis folate in the gastrointestinal tract.

Taken together, the presence of these genes related to EPS, LTA, PG, and folate biosynthesis in the genome of RGW1 provides a clue to the underlying mechanisms of the anti-inflammatory effects of both RGW1 and its metabolites in LPS-reduced mice. Further studies will aim to explore the exact molecules participating in the immunomodulatory effect of RGW1, as well as these molecules associated probiotic properties *in vivo*, especially the regulation of gut microbiota will be explored.

## 5 Conclusion

We isolated a novel *L. reuteri* strain from healthy calf feces, and systematically characterized the probiotic properties and disclosed the draft genome. The strain RGW1 showed various probiotic properties including tolerance to acid and bile salt, survival in simulated gastric and intestinal fluid, adherence to Caco-2 cells, antipathogenic and immunomodulatory effects, and carried related genes related. In addition, RGW1 could *de novo* biosynthesize L-lysine, folate, cobalamin, and reuterin. This study provides a considerable insight into the probiotic functions of *L. reuteri* RGW1 and the corresponding molecular mechanisms, thereby contributing to its application as a probiotic in antipathogen and host health regulation.

## Data availability statement

The data presented in the study are deposited in the NCBI repository, accession number PRJNA872372.

## Ethics statement

The animal study was reviewed and approved by Animal Care and Use Committee of Zhejiang Provincial Hospital of Chinese Medicine (Hangzhou, China, 60136-38385).

## Author contributions

JW designed the experiments. KH and WS performed the experiments. KH, BY, and JW analyzed the data. KH and JW wrote and revised the main manuscript. All authors contributed to the article and approved the submitted version.
