# Supplementary material for: The probiotic and immunomodulation effects of Limosilactobacillus reuteri RGW1 isolated from calf feces
Source: Front Cell Infect Microbiol. 2023 Jan 12;12:1086861. doi: 10.3389/fcimb.2022.1086861 (PMC9879569; doi:10.3389/fcimb.2022.1086861)
Supplement: Supplementary file 1 [file DataSheet_1.docx]

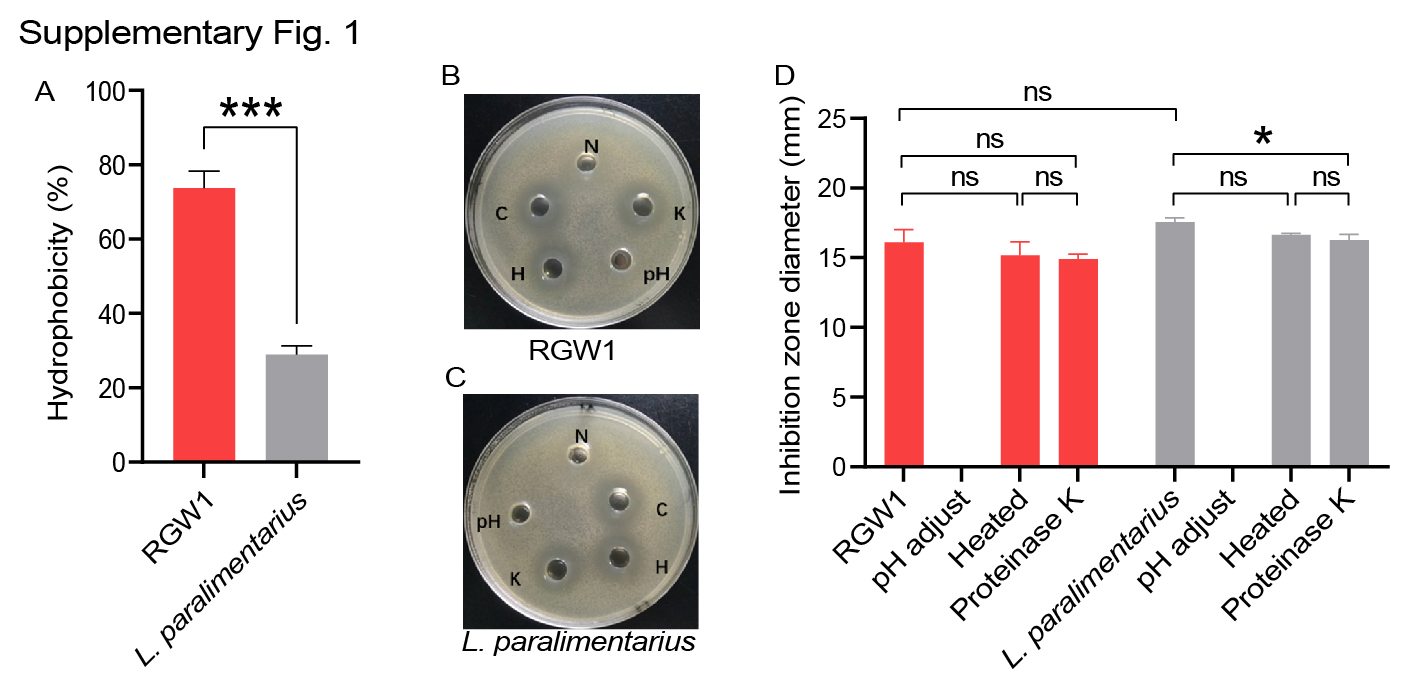


**Supplementary Figure 1.** Comparative analysis of potential probiotic properties between *L. reuteri* RGW1 and *L. paralimentarius*. **A** The hydrophobicity (%) of *L. reuteri* RGW1 and *L. paralimentarius.* **B-C** The representative images of the anti *E. coli* F5 activities of RGW1 (B) and *L. paralimentarius* (C) using agar diffusion method (In the images, N: no treatment, C: control which treated with *L. reuteri* RGW1 or *L. paralimentarius*, pH: pH adjustment, H: heating treatment, K: treated with proteinase K). **D** The result of the anti *E. coli* F5 activities of *L. reuteri* RGW1 and *L. paralimentarius* indicated by the diameters of inhibition zone, after different pretreatments. * *P <* 0.05; *** *P <* 0.001; ns, not significant.

**Supplementary Table S1.** Antimicrobial agents and associated interpretative zone diameters for disc diffusion antibiotic susceptibility testing.

| Antibiotic agent | Disc conc.(μg) | interpretative zone diameters(mm)^a^ | | |
| --- | --- | --- | --- | --- |
|  |  | R | MS | S |
| Cefazolin | 30 | ≤15 | 16-17 | ≥18 |
| penicillin G | 10 | ≤19 | 20-27 | ≥28 |
| Erythromycin | 15 | ≤13 | 14-17 | ≥18 |
| Amoxicillin | 30 | ≤18 | 19-20 | ≥21 |
| Ampicillin | 10 | ≤12 | 13-15 | ≥16 |
| Tetracycline | 30 | ≤14 | 15-18 | ≥19 |
| Chloramphenicol | 30 | ≤13 | 14-17 | ≥18 |

^a^ Susceptibility expressed as R(resistant), MS(moderately susceptible), or S(susceptible)

**Supplementary Table S2.** Primers used to detect the virulence genes.

| Virulence genes and products | Primers | Sequence (5'-3') | Product (bp) | Tm℃ |
| --- | --- | --- | --- | --- |
| *gelE* (Gelatinase) | gelE-F | ACCCCGTATCATTGGTTT | 419 | 56 |
|  | gelE-R | ACGCATTGCTTTTCCATC |  |  |
| *cylA* (Cytolysin) | cylA-F | TGGATGATAGTGATAGGAAGT | 517 | 57 |
|  | cylA-R | TCTACAGTAAATCTTTCGTCA |  |  |
| *esp* (Enterococcal surface protein) | esp-F | TTGCTAATGCTAGTCCACGACC | 933 | 62 |
|  | esp-R | GCGTCAACACTTGCATTGCCGAA |  |  |
| *efaAfs* (Cell wall adhesins) | efaAfs-F | GACAGACCCTCACGAATA | 705 | 52 |
|  | efaAfs-R | AGTTCATCATGCTGTAGTA |  |  |
| *hyl* (hyaluronidase) | HYLn1 | ACAGAAGAGCTGCAGGAAATG | 276 | 56 |
|  | HYLn2 | GACTGACGTCCAAGTTTCCAA |  |  |
| *asa* (aggregation substance) | ASA11 | GCACGCTATTACGAACTATGA | 375 | 56 |
|  | ASA12 | TAAGAAAGAACATCACCACGA |  |  |
| *hdc* (Histidine decarboxylase) | JV16HC | AGATGGTATTGTTTCTTATG | 367 | 52 |
|  | JV17HC | AGACCATACACCATAACCTT |  |  |
| *ace* (adhesion of collagen) | ACE-F | GAATTGAGCAAAAGTTCAATCG | 1008 | 55 |
|  | ACE-R | GTCTGTCTTTTCACTTGTTTC |  |  |
| *tdc* (Tyrosine decarboxylase) | P2-for | GAYATNATNGGNATNGGNYTNGAYCARG | 924 | 52 |
|  | P1-rev | CCRTARTCNGGNATAGCRAARTCNGTRTG |  |  |
| *odc* (Ornithine decarboxylase) | odc-3 | GTNTTYAAYGCNGAYAARACNTAYTTYGT | 1446 | 52 |
|  | odc-16 | ATNGARTTNAGTTCRCAYTTYTCNGG |  |  |

**Supplementary Table S3.** Lysine biosynthesis associated genes.

| Gene ID | KO ID | KO_EC ID |
| --- | --- | --- |
| Lr_GM000031 | K01439 | 3.5.1.18 |
| Lr_GM000387 | K00133 | 1.2.1.11 |
| Lr_GM000388 | K00841 | 2.6.1.- |
| Lr_GM000389 | K00215 | 1.17.1.8 |
| Lr_GM000390 | K01714 | 4.3.3.7 |
| Lr_GM000392 | K00674 | 2.3.1.117 |
| Lr_GM000393 | K01586 | 4.1.1.20 |
| Lr_GM000394 | K00928 | 2.7.2.4 |
| Lr_GM000395 | K01778 | 5.1.1.7 |
| Lr_GM000552 | K00841 | 2.6.1.- |
| Lr_GM000687 | K01439 | 3.5.1.18 |
| Lr_GM000747 | K01439 | 3.5.1.18 |
| Lr_GM000972 | K00841 | 2.6.1.- |
| Lr_GM002004 | K00841 | 2.6.1.- |

**Supplementary Table S4.** Folate biosynthesis associated genes.

| Gene ID | KO ID | KO_EC ID |
| --- | --- | --- |
| Lr_GM000118 | K01633 | 4.1.2.25 |
| Lr_GM000119 | K00950 | 2.7.6.3 |
| Lr_GM000120 | K01495 | 3.5.4.16 |
| Lr_GM000121 | K11754 | 6.3.2.12, 6.3.2.17 |
| Lr_GM000123 | K00796 | 2.5.1.15 |
| Lr_GM000162 | K06949 | 3.6.1.- |
| Lr_GM000231 | K00287 | 1.5.1.3 |
| Lr_GM001572 | K11754 | 6.3.2.12, 6.3.2.17 |

**Supplementary Table S5.** Cobalamin and reuterin biosynthesis associated genes.

| Gene ID | KO ID | KO_EC_ID | Gene name |
| --- | --- | --- | --- |
| Lr_GM000480 | K01699 | 4.2.1.28 | pduC |
| Lr_GM000481 | K13919 | 4.2.1.28 | pduD |
| Lr_GM000482 | K13920 | 4.2.1.28 | pduE |
| Lr_GM000504 | K02224 | 6.3.5.9, 6.3.5.11 | cobB-cbiA |
| Lr_GM000505 | K02227 | 6.3.1.10 | cbiB, cobD |
| Lr_GM000506 | K06042 | 5.4.99.61, 5.4.99.60 | cobH-cbiC |
| Lr_GM000507 | K02188 | 2.1.1.195 | cbiD |
| Lr_GM000508 | K03399 | 2.1.1.289 | cbiE |
| Lr_GM000509 | K02191 | 2.1.1.196 | cbiT |
| Lr_GM000510 | K05936 | 2.1.1.133, 2.1.1.271 | cobM, cbiF |
| Lr_GM000511 | K02189 | 3.7.1.12 | cbiG |
| Lr_GM000512 | K05934 | 2.1.1.131 | E2.1.1.131, cobJ, cbiH |
| Lr_GM000513 | K05895 | 1.3.1.54, 1.3.1.106 | cobK-cbiJ |
| Lr_GM000514 | K13542 | 2.1.1.107, 4.2.1.75 | cobA-hemD |
| Lr_GM000515 | K02190 | 4.99.1.3 | cbiK |
| Lr_GM000516 | K03394 | 2.1.1.130, 2.1.1.151 | cobI-cbiL |
| Lr_GM000517 | K02007 | -- | cbiM |
| Lr_GM000518 | K02009 | -- | cbiN |
| Lr_GM000519 | K02008 | -- | cbiQ |
| Lr_GM000520 | K02006 | -- | cbiO |
| Lr_GM000521 | K02232 | 6.3.5.10 | cobQ, cbiP |
| Lr_GM000522 | K02304 | 1.3.1.76, 4.99.1.4 | MET8 |
| Lr_GM000523 | K02492 | 1.2.1.70 | hemA |
| Lr_GM000524 | K01749 | 2.5.1.61 | hemC, HMBS |
| Lr_GM000525 | K01698 | 4.2.1.24 | hemB, ALAD |
| Lr_GM000526 | K01845 | 5.4.3.8 | hemL |
| Lr_GM000527 | K02231 | 2.7.1.156, 2.7.7.62 | cobP, cobU |
| Lr_GM000528 | K02233 | 2.7.8.26 | E2.7.8.26, cobS, cobV |
| Lr_GM000529 | K02226 | 3.1.3.73 | cobC, phpB |
| Lr_GM000531 | K00768 | 2.4.2.21 | E2.4.2.21, cobU, cobT |
